# Impact of Polypharmacy and P-Glycoprotein- and CYP3A4-Modulating Drugs on Safety and Efficacy of Oral Anticoagulation Therapy in Patients with Atrial Fibrillation

**DOI:** 10.1007/s10557-019-06907-8

**Published:** 2019-09-13

**Authors:** Ralf E. Harskamp, Martina Teichert, Wim A. M. Lucassen, Henk C. P. M. van Weert, Renato D. Lopes

**Affiliations:** 1 Department of General Practice, Amsterdam UMC, Amsterdam Public Health and Amsterdam Cardiovascular Sciences Research Institutes, Academic Medical Center, University of Amsterdam, Meibergdreef 9, 1105 AZ Amsterdam, The Netherlands; 2 Duke Clinical Research Institute, Duke University Medical Center, Durham, NC USA; 3 Department of Clinical Pharmacy and Toxicology, Leiden University Medical Centre, Leiden, The Netherlands

**Keywords:** Polypharmacy, Drug-drug interactions, DOAC, Warfarin, Atrial fibrillation

## Abstract

**Purpose:**

To study whether polypharmacy or drug–drug interactions have differential effect on safety and efficacy in patients treated with direct oral anticoagulants (DOACs) versus warfarin.

**Methods:**

We performed a systematic review and meta-analysis of studies that randomized patients with atrial fibrillation to DOACs or warfarin stratified by the number of concomitant drugs. Outcomes included stroke or systemic embolism (SE), all-cause mortality, major bleeding, and intracranial hemorrhage. Risk ratios (RR) were calculated and Mantel-Haenszel random effects were applied.

**Results:**

Two high-quality studies were eligible, including 32,465 participants who received apixaban, rivaroxaban, or warfarin, with a median follow-up of 1.9 years. Of participants, 29% used < 5 drugs, 55% used 5–9 drugs, and 16% used ≥ 10 drugs. Drugs interacting with DOACs (P-glycoprotein/CYP3A4) were used by 6460 (20%) of patients. Patients with higher number of drugs (0–4 vs 5–9 vs ≥ 10) had higher rates of mortality (5.8%, 7.9%, 10.0%) and major bleeding (3.4%, 4.8%, 7.7%). Comparative efficacy or safety of DOACs versus warfarin was not affected by polypharmacy status or P-glycoprotein/CYP3A4 inhibitor use. However, the presence of polypharmacy (*p* = 0.001) or glycoprotein/CYP3A4-modulating drugs (*p* = 0.03) was correlated with increased risk of major bleeding when compared with warfarin. Overall, DOAC use was associated with a lower risk of stroke/SE (RR, 0.84; 95%CI, 0.74–0.94), all-cause mortality (RR, 0.91; 95%CI, 0.84–0.98), and intracranial hemorrhage (RR, 0.51; 95%CI, 0.38–0.70) compared with warfarin.

**Conclusions:**

DOACs were more effective than warfarin, and at least as safe. Polypharmacy was associated with adverse outcomes and attenuated the advantage in risk of major bleeding among rivaroxaban users, particularly in the presence of P-glycoprotein/CYP3A4-modulating drugs.

**Electronic supplementary material:**

The online version of this article (10.1007/s10557-019-06907-8) contains supplementary material, which is available to authorized users.

## Introduction

Atrial fibrillation (AF) is a common cardiac arrhythmia, which prevalence increases with age. Currently, the lifetime risk for developing AF is > 30% for people age 55 and older [1]. AF may result in unfavorable health outcomes that include a fivefold increased risk of stroke and systemic embolism, hospitalization, and impaired quality of life [2]. To reduce the risk of thromboembolic complications, most patients with AF require oral anticoagulants. However, when initiating therapy, physicians often face patients who also require drugs for co-existing chronic medical conditions which often result in polypharmacy and unwanted drug–drug interactions [3]. Prior studies have shown that the presence of polypharmacy, which is usually defined as 5 or more drugs, translates into a higher risk of death and bleeding complications in patients with AF [4, 5]. While drug–drug interactions may account for these complications, the regimen complexity that accompanies polypharmacy may also negatively affect adherence [6]. Currently, patients with AF who require anticoagulant therapy either receive vitamin K antagonists (of which “warfarin” is typically used in most countries) or non-vitamin K oral anticoagulants (DOACs). The latter were introduced about a decade ago and are more user-friendly than warfarin. Moreover, unlike warfarin, DOACs are also thought to have fewer drug–drug interactions and interactions with food. However, interacting pathways exist for DOACs and a number of commonly used drugs and include the permeability glycoprotein (P-gp) efflux transporter protein and/or cytochrome P (CYP) 450 3A4 enzymes [7]. Despite the common use of oral anticoagulants and frequent occurrence of polypharmacy, data assessing the safety and efficacy of DOACs compared with warfarin are sparse. This is of interest, as one may suspect that polypharmacy, particularly with P-gp- and CYP3A4-interacting drugs, could lead to differential response to anticoagulation therapy. As such, we studied the following research questions: (1) Does the number of concomitant drugs in AF patients treated with oral anticoagulant maintenance therapy increase the risk for adverse outcomes, and (2) Does this risk differ between DOACs and a vitamin K antagonist in the presence or absence of polypharmacy and/or interacting drugs?

## Methods

The Preferred Reporting Items for Systematic Reviews and Meta-Analyses (PRISMA) guidelines were used to undertake this review.

### Data Sources and Searches

We performed a Medline/PubMed search from January 2009 until the search date February 4, 2019. We searched for studies written in English and conducted in humans of 18+ years of age. We used keywords for atrial fibrillation in combination with keywords for vitamin K antagonists and DOACs. (The search can be found in the supplemental data document.) We also consulted the contact persons for the included trials to provide additional information.

### Study Selection

Two investigators (REH, RDL) identified potentially eligible studies. We used an online systematic review platform (Rayyan, Qatar Computing Research Institute, Doha, Qatar) [8]. The search was restricted to publications concerning human research, age > 18, and written in English, Dutch, German, or Portuguese. We applied the following inclusion criteria: (1) original data studies presenting follow-up data on stroke and major bleeding; (2) involving polypharmacy and/or drug–drug interaction (P-gp or CYP3A4); polypharmacy referred to the use of 5 or more drugs; (3) involving a randomized comparison between a DOAC (apixaban, dabigatran, rivaroxaban, or edoxaban) versus a vitamin K antagonist (warfarin, coumadin, etc.).

### Study Population

We included studies with adult populations that were randomized to a DOAC or vitamin K antagonist for non-valvular atrial fibrillation, in which the comparative safety and efficacy were stratified by the number of concomitant drugs.

### Outcomes of Interest

The outcomes of interest included efficacy and safety endpoints using study-reported definitions. For efficacy, we included stroke or systemic embolism and all-cause mortality. For safety, we included the following bleeding outcomes: major bleeding, intracranial hemorrhage, and clinically relevant non-major (CRNM) bleeding. Finally to balance therapeutic benefits with risks of associated treatment, we provided a joint estimate or “net clinical benefit” [9]. This outcome consisted of a composite of stroke or systemic embolism, major bleeding, or (vascular) death.

### Data Extraction and Quality Assessment

One investigator (REH) extracted data elements from each study, with a second investigator (WAML) independently reviewing these data for accuracy. The quality of the studies was assessed using the Cochrane Risk of Bias tool for assessing the risk of bias in randomized studies.

### Data Synthesis and Analysis

The extracted data on study and patient characteristics, outcome measures, and follow-up information of the included studies were displayed in tables. Data were reported on the association of polypharmacy on clinical outcomes, as well as the comparative safety and efficacy of DOACs versus vitamin K antagonists in the studied populations. To obtain comparative outcome data on the number of concomitant drugs (< 5, 5–9, and ≥ 10), we obtained additional data from the ARISTOTLE trial as the original manuscript only provided data on (< 6, 6–8, and > 8). We also obtained data on other outcomes than on major bleeding for P-gp- and CYP3A4-interacting drugs. Clinical outcomes data were presented as relative risk and 95% confidence intervals, and we applied the empirical Bayes methods to account for heterogeneity. We compared differences in relative risks for safety and efficacy of DOACs versus vitamin K antagonist for patients stratified according to (1) the presence of polypharmacy and (2) P-gp/CYP3A4-modulating drugs. All analyses were performed with Review Manager (RevMan version 5.3, The Cochrane Nordic Collaboration, Copenhagen, Denmark) and JASP Stats (JASP version 0.10.2, University of Amsterdam, The Netherlands).

## Results

### Search Results

**Fig. 1:**
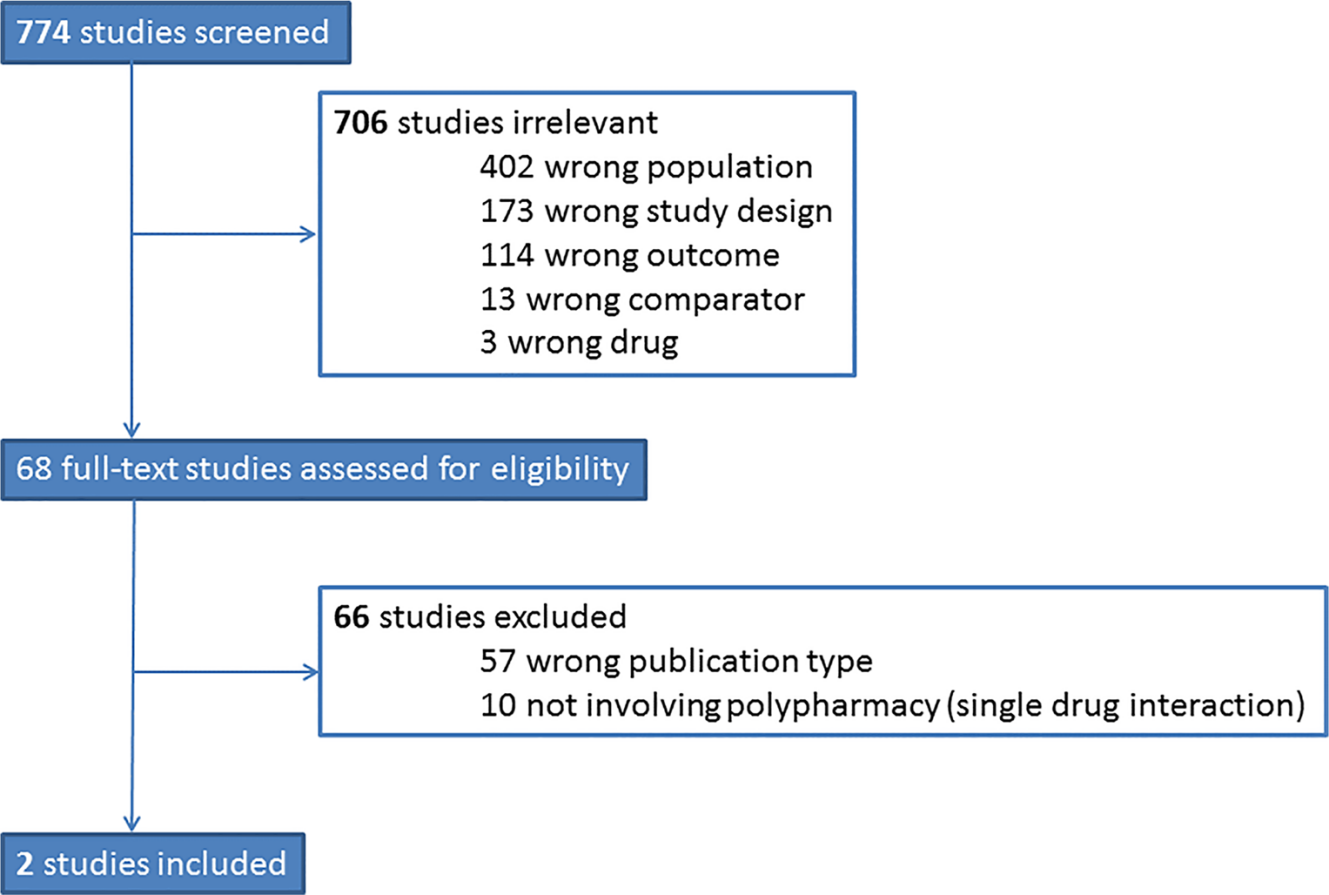
Flowchart of search

Our search included 774 studies of which we assessed 68 in full-text. Of those, 2 studies met the inclusion criteria of our study. These studies were post hoc analyses from the ARISTOTLE (apixaban versus warfarin) and ROCKET-AF (rivaroxaban versus warfarin) trials [10, 11]. The flowchart of our search strategy and reasons for exclusions can be found as Fig. 1.

### Quality Assessment

As shown in Table 1, the two studies were well designed and conducted, which renders the risk of bias overall low. Given that both studies involved post hoc analyses of randomized trials, we assessed for additional biases. A potential source of bias lies in the assumption that patients with polypharmacy at baseline maintained this status throughout the study duration and vice versa. Moreover, the duration of P-gp- and/or CYP3A4-interacting medication use was not captured. Another source of bias lies in insufficient statistical power, given that we assessed subgroup analyses within populations which sample sizes were not powered for polypharmacy.

**Table 1:** Quality assessment of the studies included

	ARISTOTLE (apixaban vs warfarin)	ROCKET-AF (rivaroxaban vs warfarin)
Random sequence generation	⊕	⊕
Allocation concealment	⊕	⊕
Selective reporting	⊕	⊕
Other bias*	Unclear	Unclear
Blinding of participants and personnel	⊕	⊕
Blinding of outcome assessment	⊕	⊕
Incomplete outcome data	⊕	⊕

*A post hoc, non-pre-specified analysis

### Study Characteristics

The two included studies comprised 32,465 participants, who were recruited in the Americas, Europe, and Asia between 2006 and 2009 for ROCKET-AF (*n* = 14,264) and 2006 and 2011 for ARISTOTLE (*n* = 18,201) with a median follow-up duration of 1.9 years [10, 11]. The key inclusion and exclusion criteria for the ARISTOTLE and ROCKET-AF trials can be found in Table S2. Most notably, in the ARISTOTLE and ROCKET-AF trial, the use of strong CYP3A4 inhibitor or inducer was an exclusion criterion for trial participation. This included drugs such as azole antifungals, macrolide antibiotics, and protease inhibitors.

### Patient Characteristics

**Fig. 2:**
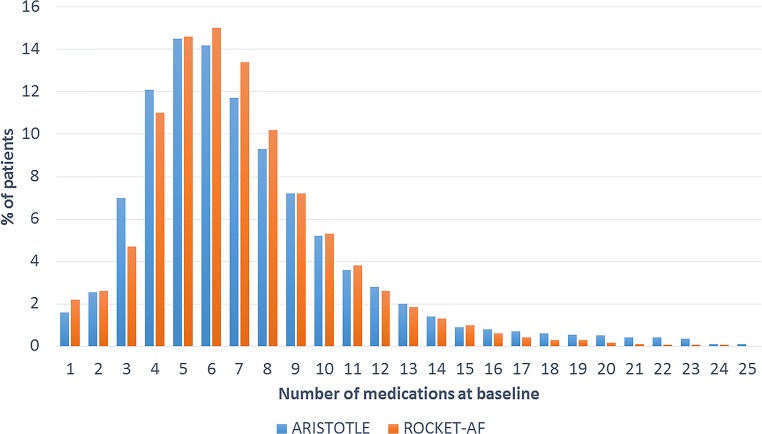
Use of concomitant medications in ARISTOTLE and ROCKET-AF

Polypharmacy was present in over two-thirds of patients enrolled in the ARISTOTLE or ROCKET-AF trials. The distribution of the number of concomitant drugs is displayed in Fig. 2. Of the participants, 29% used fewer than 5 drugs, 55% used 5–9 drugs, and 16% used ≥ 10 drugs. Randomization to DOAC versus warfarin was evenly distributed among patients with and without polypharmacy. Patient characteristics were markedly different depending on the presence of polypharmacy, as shown in Table 2. Patients with polypharmacy tended to be older and more often had comorbidities, such as diabetes, COPD, and (cardio) vascular disease, than those without polypharmacy. Data from ARISTOTLE suggest that patients with polypharmacy more often had a history of anemia or prior bleeding. The presence of osteoporosis, falls, and prior non-traumatic fractures was also higher, suggesting that “frailty” was more common in patients with polypharmacy compared with study participants without polypharmacy. A total of 6460 (20%) patients used at least one drug with a combined inhibition of P-gp and CYP3A4 at time of trial enrollment, which was more common in patients with polypharmacy (23% vs 15%). Combined inhibitors included amiodarone, diltiazem, verapamil, quinidine, ranolazine, felodipine, erythromycin, or azithromycin.

**Table 2:** Baseline patient characteristics and comorbidity of ARISTOTLE and ROCKET-AF stratified by polypharmacy status*

	ARISTOTLE (*n* = 18,201)		ROCKET-AF (*n* = 14,264)	
	Polypharmacy	No polypharmacy	Polypharmacy	No polypharmacy
Patients (number)	13,932 (77%)	4269 (23%)	9163 (64%)	5101 (36%)
Age (years)	70 (±9)	68 (±10)	73 (66, 78)	71 (64, 77)
Male	8831 (63%)	2954 (69%)	5444 (59%)	3160 (62%)
BMI	30 (±6)	28 (±5)	29 (26–33)	27 (24–31)
CHADS2-score (≥ 3)	4661 (34%)	841 (20%)	8085 (88%)	4317 (85%)
Prior MI	2287 (16%)	298 (7%)	1912 (21%)	556 (11%)
Congestive heart failure	4498 (32%)	1043 (24%)	6071 (66%)	2837 (56%)
Prior stroke/TIA	2249 (16%)	577 (14%)	4363 (48%)	3447 (68%)
Peripheral artery disease	781 (6%)	103 (3%)	651 (7%)	188 (4%)
Diabetes mellitus	4117 (30%)	430 (10%)	4509 (49%)	1186 (23%)
Hypertension	12,422 (89%)	3494 (82%)	8570 (94%)	4340 (85%)
Creatinine clearance (mL/min)	79 (±33)	81 (±30)	67 (51, 86)	68 (54, 87)
COPD	1718 (12%)	232 (6%)	1198 (13%)	299 (6%)
Sleep apnea	934 (7%)	79 (2%)	–	–
Dementia	87 (<1%)	9 (<1%)	–	–
History of anemia	1121 (8%)	124 (3%)	–	–
Prior bleeding	2580 (19%)	460 (11%)	–	–
Osteoporosis	887 (6%)	83 (2%)	–	–
Falls within 1 year	668 (5%)	85 (2%)	–	–
Prior non-traumatic fracture	908 (7%)	166 (4%)	–	–
Medications				
Randomized to DOAC	7022 (50.4%)	2098 (49.1%)	4590 (50%)	2541 (50%)
≥ 1 combined P-gp and CYP3A4 inhibitor	2732 (24%)	1128 (16%)	1905 (21%)	695 (14%)

*Polypharmacy status for a patient was defined as 5 or more drugs in concomitant use at baseline

### Polypharmacy and Adverse Clinical Outcomes

**Fig. 3:**
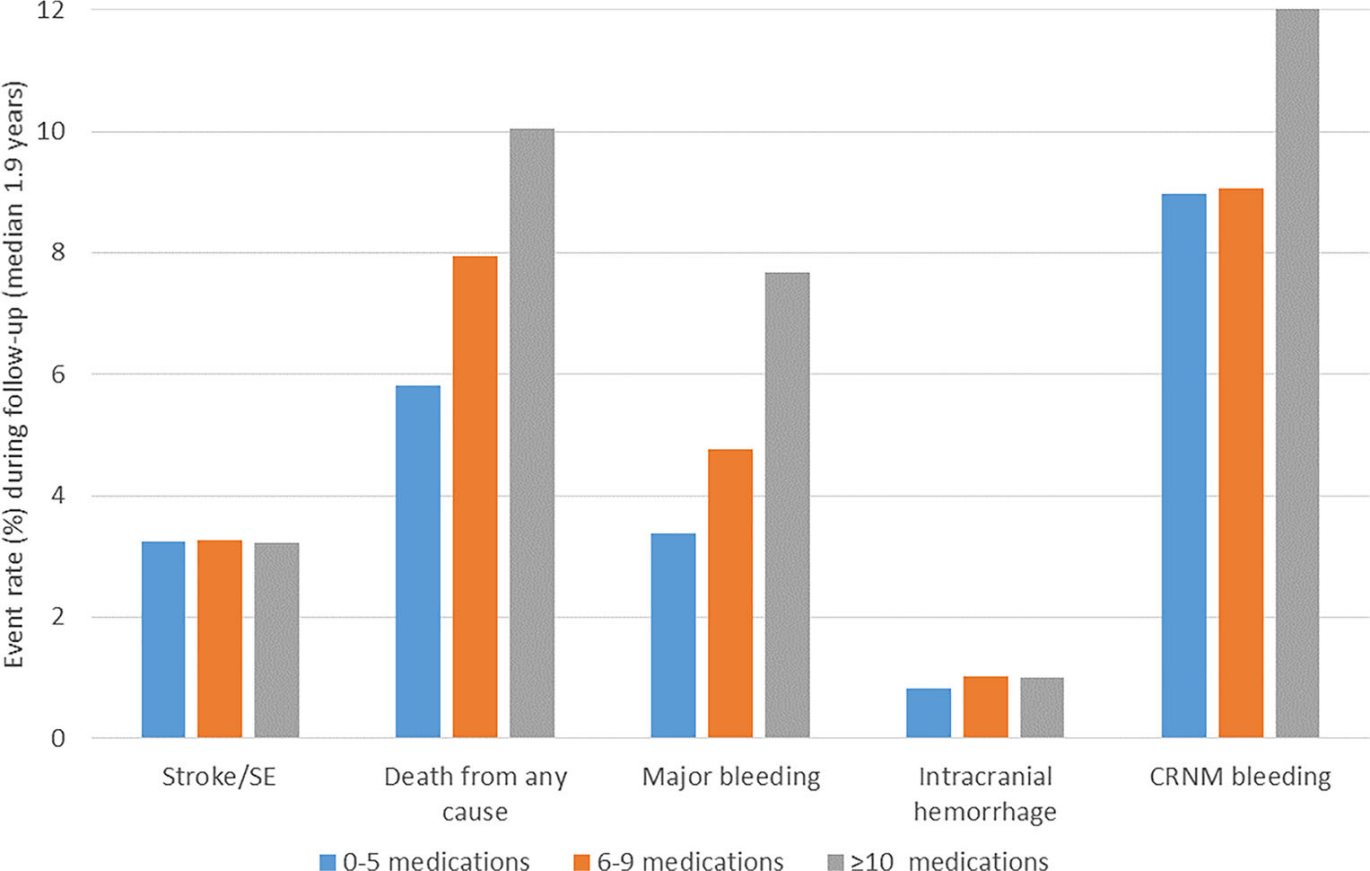
Event rate (%) during follow-up stratified by number of concomitant medications among trial participants (both DOAC and warfarin)

Figure 3 displays the event rates stratified by the number of concomitant drugs. Patients with higher number of concomitant drugs (0–4 vs 5–9 vs ≥ 10 drugs) had higher rates of mortality (5.8%, 7.9%, 10.0%), major bleeding (3.4%, 4.8%, 7.7%), and CRNM bleeding events (9.0%, 9.1%, 12.2%). The rates of stroke/SE (3.2%, 3.3%, 3.2%) in the studied population of moderate- to high-stroke-risk patients were comparable, as were the rates of intracranial hemorrhage (0.8%, 1.0%, 1.0%). These trends were observed for patients using DOACs as well as warfarin.

### Comparative Safety and Efficacy of DOACs Versus Warfarin Stratified by Polypharmacy

Table 3 summarizes the relative safety and efficacy of DOACs versus warfarin stratified by the presence of polypharmacy. As shown, polypharmacy did not interact with the comparative safety or efficacy of DOACs versus VKA. DOAC use was associated with consistent lower risk of stroke/SE (RR, 0.84 [0.74–0.94]), all-cause mortality (RR, 0.91; 95%CI, 0.84–0.98), intracranial hemorrhage (RR, 0.51; 95%CI, 0.38–0.70), and net clinical benefit (RR, 0.87; 95%CI, 0.82–0.93), when compared with warfarin use. When evaluating DOACs separately, as shown in Table S3, we found an interaction for polypharmacy with major bleeding for users of rivaroxaban (*p* = 0.001), but not for apixaban (*p* = 0.14) when compared with that for subjects randomized to warfarin.

**Table 3:** Efficacy and safety outcomes for DOAC and warfarin use, stratified by polypharmacy status

	Polypharmacy	DOAC	Warfarin	RR	*I*^2^	*P* value
Stroke	Yes	339 (2.9%)	410 (3.6%)	0.90 [0.70–1.10]	0%	0.31
	No	142 (3.1%)	161 (3.4%)	0.82 [0.70–0.94]		
Death from any cause	Yes	931 (8.0%)	1012 (8.8%)	0.92 [0.84–0.99]	0%	0.82
	No	254 (5.5%)	289 (6.1%)	0.90 [0.75–1.04]		
Major bleeding	Yes	603 (5.2%)	652 (5.7%)	0.94 [0.64–1.24]	85%	0.15
	No	119 (2.6%)	196 (4.1%)	0.61 [0.29–0.93]		
Intracranial hemorrhage	Yes	80 (0.7%)	156 (1.4%)	0.53 [0.18–0.87]	69%	0.99
	No	27 (0.6%)	50 (1.1%)	0.53 [0.11–0.94]		
CRNM bleed	Yes	1081 (9.3%)	1179 (10.3%)	0.85 [0.56–1.14]	90%	0.62
	No	422 (9.1%)	416 (8.8%)	0.96 [0.64–1.27]		
Net clinical benefit *	Yes	1265 (10.9%)	1415 (12.3%)	0.88 [0.82–0.95]	4%	0.43
	No	343 (7.4%)	417 (8.9%)	0.83 [0.71–0.95]		

*ARISTOTLE, stroke/SE/major bleed/death; *ROCKET*, stroke/SE/fatal bleed/vascular death

### Comparative Safety and Efficacy of DOACs Versus Warfarin Stratified by Concomitant P-Gp and CYP3A4 Inhibitor Use

The relative safety and efficacy of DOACs and warfarin stratified by the use of ≥ 1 combined P-gp- and CYP3A4-interacting medication is shown in Table 4. Overall, we did not find an association for any of the outcomes. When assessing individual DOACs (Table S4), we found a significant interaction with major bleeding for rivaroxaban versus warfarin (*p* = 0.03), in which the combination of rivaroxaban with ≥ 1 combined P-gp and CYP3A4 inhibitor was associated with a higher bleeding risk compared with warfarin (RR, 1.37 [1.01–1.85], *p* = 0.04). No interaction was found between apixaban versus warfarin in the presence or absence of interacting drugs (*p* = 0.51).

**Table 4:** Clinical outcomes in patients with DOAC or warfarin according to the use of ≥ 1 combined P-glycoprotein- and CYP3A4-interacting medication

	≥ 1 P-gp/CYP3A4	DOAC	Warfarin	RR	*I*^2^	*P* value
Stroke	Yes	3.0% (3224)	3.1% (3236)	0.96 [0.69–1.22]	0%	0.36
	No	3.0% (384)	3.1% (470)	0.82 [0.71–0.93]		
Death from any cause	Yes	6.9% (593)	7.4% (622)	0.93 [0.83–1.04]	0%	0.83
	No	7.2% (943)	8.0% (1032)	0.93 [0.84–1.00]		
Major bleeding	Yes	5.2% (168)	5.0% (161)	1.03 [0.57–1.49]	91%	0.50
	No	4.3% (554)	5.3% (687)	0.82 [0.41–1.23]		
Intracranial hemorrhage	Yes	0.7% (23)	1.5% (51)	0.44 [0.17–0.70]	29	0.59
	No	0.6% (84)	1.2% (155)	0.53 [0.34–0.72]		
CRNM bleed	Yes	9.9% (318)	9.6% (309)	0.91 [0.62–1.19]	90%	0.67
	No	9.1% (1185)	9.9% (1286)	0.86 [0.59–1.13]		
Net clinical benefit *	Yes	9.9% (319)	11.3% (366)	0.88 [0.75–1.00]	0%	0.93
	No	9.9% (1289)	11.3% (1466)	0.87 [0.81–0.93]		

*ARISTOTLE, stroke/SE/major bleed/death; *ROCKET*, stroke/SE/fatal bleed/ vascular death

#### Discussion

Polypharmacy is common in patients with atrial fibrillation and presents a challenge given the risk of medication interactions which in turn may affect the safety and efficacy of antithrombotic therapy. In this study, we found that polypharmacy was present in over two-thirds of the study population and DOAC-interacting drugs were present in about a quarter of patients. The patients with polypharmacy tended to be sicker with more comorbidities, along with a higher percentage of interacting medications. As a result, we found that the frequencies of bleeding events and mortality, but not of stroke and systemic embolism, were increased with increasing number of concomitant drugs. The presence of polypharmacy as well as DOAC-interacting drugs appears to attenuate the advantage of rivaroxaban over warfarin for major bleeding, but not for apixaban, whereas for both DOACs, a more favorable profile is observed for other clinical outcomes, including stroke, all-cause mortality, and net clinical benefit.

### DOACs and Warfarin

Prophylaxis of ischemic stroke with warfarin has been the gold standard of care for decades [12, 13]. The success of warfarin lies in preventing thrombosis that causes stroke and systemic embolism and in low direct costs. However, warfarin also has a narrow therapeutic range that requires frequent blood testing and dosing adjustment [12]. Furthermore, interactions of warfarin are numerous, not only for prescription and over-the-counter drugs but also for vitamins, herbal products, and food [14]. Over the last decade, DOACs have shown to have a number of pharmacologic advantages over warfarin, including rapid onset/offset of action, fewer food and drug interactions, and a consistent antithrombotic effect that allows for fixed dosing without the need for coagulation status monitoring [13]. Moreover, in clinical trials as well as real-world data, DOACs are consistently found to be at least as effective and safe as warfarin [15]. For this reason, the latest update on AF treatment guidelines now recommends DOACs as the preferred alternative to warfarin for reducing the risk of stroke [16]. However, physicians should be aware that long-term effects as well as the effects of polypharmacy and specific concomitant drugs have not been as extensively studied in DOACs yet.

### Polypharmacy

Our findings corroborate with prior studies, in which polypharmacy was associated with excess risk of mortality and bleeding complications, but not stroke in the setting of AF [5]. In AFFIRM, the presence of polypharmacy conferred 30% excess relative risk for cardiovascular death, after adjusting for age and other comorbidity factors [5]. These data suggest different pathophysiological patterns, and the presence of polypharmacy may help identify patients with more frailty. Careful assessment of the appropriateness of prescription is warranted, with a growing body of evidence showing that the process of “deprescribing” is an effective strategy to reduce the number of inappropriate drugs and consequently the number of adverse drug reactions and adverse events [5, 17]. Such a patient-centered prescribing process would seem appropriate, particularly in the setting of AF. However, to our knowledge, high-quality description trials have not been performed to assess whether these strategies would indeed result in optimized clinical outcomes in patients with AF.

### DOAC-Related Drug Interactions

Given the relatively lower number of (known) drug interactions of DOACs, it would seem appropriate that most patients with polypharmacy could be better treated with DOACs instead of warfarin. However, one should be aware of possible DOAC interactions, some being common cardiovascular medications [5]. To help clinicians, the European Heart Rhythm Association has recently issued a practical guide on the use of DOACs in patients with AF [13]. In this guideline document, the authors make specific recommendations on DOAC choice in combination with polypharmacy and multiple interacting drugs. Important cardiovascular medications with potential interactions that are mentioned in this document are as follows: amiodarone, verapamil, and diltiazem (apixaban) and ticagrelor (for dabigatran). Drug–drug interaction with other common medications, such as antibiotics (rifampicin, clarithromycin, erythromycin) and fungostatics (i.e., ketoconazole), is also listed. It is important to realize that the authors made many of their recommendations based on the results from pharmacokinetic studies, as data from clinical outcome–based studies to support these mechanistic studies are limited.

One of the few studies that assessed the clinical effects of DOACs with concomitant interacting drugs was obtained from a population-based cohort from Taiwan [18]. In this study, involving over 90,000 patients, the authors studied major bleeding risk in patients with DOACs with or without concomitant use of 12 commonly prescribed P-gp competitors and/or CYP3A4 inhibitors. The authors found that drugs within these two metabolic pathways were associated with a higher bleeding risk. When assessing the impact of single drug, the concurrent use of amiodarone, fluconazole, rifampin, and phenytoin was associated with (clinically) significant increase in major bleeding risk. Other drugs with interaction profiles based on pharmacokinetic studies (such as diltiazem, verapamil, and ketoconazole) were not associated with increased bleeding risk. Moreover, the combined use of a DOAC with atorvastatin, digoxin, clarithromycin, or erythromycin was associated with a reduced risk for major bleeding. These findings underline that pharmacokinetic data may not always line up with associations observed in clinical outcome–based studies. However, overall caution is warranted for drugs that share key metabolic pathways with DOACs in regard to bleeding risk. Unfortunately, the Taiwanese study does not provide information on the impact on efficacy outcomes, such as stroke and mortality, or on the relative efficacy and safety when compared with warfarin. In that regard, the findings of our study may provide reassuring answers, as the data clearly demonstrate that in the presence of interacting drugs, the use of DOACs remains more effective than warfarin. For specific drugs, such as amiodarone and other antiarrhythmic drugs, the use of DOACs was also found to be associated with more favorable in terms of both bleeding and stroke risk when compared with warfarin [19–21].

### Limitations and Future Directions

There are a number of limitations that deserve to be mentioned. Our study relied on data from post hoc analyses from the ARISTOTLE and ROCKET-AF trials. While the randomization process for DOAC versus warfarin still upholds, we should consider these secondary analyses exploratory in nature. Moreover, the data on medication use was limited to baseline information. As such medication changes, discontinuation or initiation of new (interacting) drugs was not captured. The cut-off value of ≥ 5 drugs for polypharmacy, while commonly accepted, is arbitrary. With regard to generalizability, our findings may not apply to patients who were taking strong CYP3A4-interacting drugs (ketoconazole, erythromycin, clarithromycin, rifampin, and protease inhibitors) as these patients were not eligible for participation in the ARISTOTLE or ROCKET-AF trials. Furthermore, our findings do not extend to edoxaban and dabigatran, as they were not studied. Further exploration of the effects of polypharmacy and medication interactions of individual DOACs is warranted as observational studies suggest differences in the relative safety and efficacy between these DOACs [22–24]. In this regard, individual patient-level data analyses or direct head-to-head comparisons between safety and efficacy of the four available DOACs would be very welcome. Lastly, in our study, we ignored the possibility of unknown interacting pathways between drugs, other than the P-gp and CYP3A4 pathways. In this regard, new ways of identifying medication interactions, such as through data mining, may lead to new insights [25].

## Conclusion

Overall, in the studied population, DOACs were more effective than warfarin, and at least as safe. Polypharmacy was common among patients with atrial fibrillation requiring oral anticoagulant therapy. It was associated with adverse clinical outcomes and risk of major bleeding for DOACs, but not with other outcomes. Clinicians should be aware about the presence of polypharmacy and/or drugs with interacting pathways, such as amiodarone, as they are associated with adverse clinical outcomes and interact with the risk of major bleeding for DOACs, especially when rivaroxaban is used.

## Electronic supplementary material

ESM 1 (DOCX 35 kb)
