# Supplementary material for: Impact of Polypharmacy and P-Glycoprotein- and CYP3A4-Modulating Drugs on Safety and Efficacy of Oral Anticoagulation Therapy in Patients with Atrial Fibrillation
Source: Cardiovasc Drugs Ther. 2019 Sep 13;33(5):615–23. doi: 10.1007/s10557-019-06907-8 (PMC6904377; doi:10.1007/s10557-019-06907-8)
Supplement: Supplementary file 1 — (DOCX 35 kb) [file 10557_2019_6907_MOESM1_ESM.docx]

**Supplemental data**

**Table S1.** PubMed/Medline Database search

| **Search** | **Details** | **Hits** |
| --- | --- | --- |
| #1 | Search (((((((((factor xa[MeSH Terms]) OR direct thrombin inhibitors[MeSH Terms]) OR rivaroxaban[Title/Abstract]) OR apixaban[Title/Abstract]) OR dabigatran[Title/Abstract]) OR edoxaban[Title/Abstract]) OR direct oral anticoagulant[Title/Abstract]) OR novel oral anticoagulant[Title/Abstract]) OR DOAC[Title/Abstract]) OR DOAC[Title/Abstract] | 17540 |
| #2 | Search (((warfarin[MeSH Terms]) OR coumadin[MeSH Terms]) OR vitamin K antagonist[Title/Abstract]) OR VKA[Title/Abstract] | 20931 |
| #3 | Search (atrial fibrillation[MeSH Terms]) OR atrial fibrillation[Title/Abstract] | 73020 |
| #4 | Search ((#3) AND #1) AND #2 Filters: Publication date from 2009/01/01 to 2019/02/04; Humans; English; Adult: 19+ years | 774 |

**Table S2.** Details of included studies

| ARISTOTLE  Multicenter, double blind, double dummy | Comparison groups:   1. Apixaban 5 mg twice daily (or 2.5 mg twice daily in patients age ≥80 years, body weight <60kg, serum creatinine ≥132.6 μmol/L) 2. Dose-adjusted warfarin (target international normalized ratio, 2.0–3.0)   Inclusion criteria:  Non-valvular AF or flutter at moderate to high  risk of stroke, defined by symptomatic heart failure, left ventricular ejection fraction ≤40%, hypertension requiring medications, age ≥75, diabetes mellitus, prior stroke/TIA or systemic embolism.  Exclusion criteria:  hemodynamically significant mitral stenosis, aspirin treatment >165 mg/daily in combination with a theinopyridine, recent ischemic stroke, atrial fibrillation due to reversible causes, severe renal insufficiency (that is,  serum creatinine >221.0 μmol/L or calculated creatinine  clearance <0.42 mL/s). |
| --- | --- |
| ROCKET-AF  Multicenter, double-blind, double-dummy | Comparison groups:   1. Rivaroxaban 20 mg once daily (or 15 mg daily in patients with creatinine clearance 30–49 mL/min) 2. Dose-adjusted warfarin (target international normalized ratio, 2.0–3.0)   Inclusion criteria:  Non-valvular AF at moderate to high  risk of stroke, defined by CHADS2 risk score ≥2. Enrollment of patients with only 2 risk factors was capped at 10% for each geographic region.  Exclusion criteria:  use of a strong CYP3A4 inhibitor or inducer, prosthetic  heart valves, hemodynamically significant mitral stenosis, and creatinine clearance <30 mL/min. |

**Table S3.** Comparative safety and efficacy of apixaban or rivaroxaban versus warfarin stratified by presence of polypharmacy

|  | **DOAC** | **Polypharmacy** | **RR** | **P-value** |
| --- | --- | --- | --- | --- |
| Stroke | Apixaban | Yes | 0.76 [0.62, 0.93] | 0.35 |
|  |  | No | 0.94 [0.64, 1.38] |  |
|  | Rivaroxaban | Yes | 0.88 [0.72, 1.08] | 0.98 |
|  |  | No | 0.88 [0.67, 1.15] |  |
| Death from any cause | Apixaban | Yes | 0.91 [0.81, 1.02] | 0.61 |
|  |  | No | 0.84 [0.65, 1.09] |  |
|  | Rivaroxaban | Yes | 0.92 [0.81, 1.04] | 0.87 |
|  |  | No | 0.94 [0.76, 1.16] |  |
| Major bleeding | Apixaban | Yes | 0.74 [0.63, 0.86] | 0.14 |
|  |  | No | 0.55 [0.39, 0.78] |  |
|  | Rivaroxaban | Yes | 1.16 [0.99, 1.35] | 0.001 |
|  |  | No | 0.67 [0.50, 0.90] |  |
| Intracranial bleeding | Apixaban | Yes | 0.35 [0.24, 0.52] | 0.05 |
|  |  | No | 0.73 [0.39, 1.36] |  |
|  | Rivaroxaban | Yes | 0.77 [0.52, 1.14] | 0.10 |
|  |  | No | 0.39 [0.19, 0.81] |  |
| CRNM bleed | Apixaban | Yes | 0.69 [0.59, 0.81] | 0.44 |
|  |  | No | 0.80 [0.58, 1.11] |  |
|  | Rivaroxaban | Yes | 1.01 [0.92, 1.10] | 0.38 |
|  |  | No | 1.08 [0.94, 1.24] |  |
| Net clinical benefit | Apixaban | Yes | 0.88 [0.81, 0.96] | 0.16 |
|  |  | No | 0.76 [0.62, 0.92] |  |
|  | Rivaroxaban | Yes | 0.89 [0.79, 1.01] | 0.69 |
|  |  | No | 0.93 [0.77, 1.13] |  |

**Table S4.** Comparative safety and efficacy of apixaban or rivaroxaban versus warfarin stratified by the use of ≥1 combined P-glycoprotein and CYP3A4 interacting medication

|  | **DOAC** | **P-gp/CYP3A4** | **RR** | **P-value** |
| --- | --- | --- | --- | --- |
| Stroke | Apixaban | <1 | 0.77 [0.63-0.94] | 0.42 |
|  |  | ≥1 | 0.92 [0.62-1.38] |  |
|  | Rivaroxaban | <1 | 0.87 [0.73-1.03] | 0.56 |
|  |  | ≥1 | 0.99 [0.68-1.44] |  |
| Death from any cause | Apixaban | <1 | 0.92 [0.82-1.04] | 1.00 |
|  |  | ≥1 | 0.92 [0.82-1.04] |  |
|  | Rivaroxaban | <1 | 0.90 [0.80-1.02] | 0.42 |
|  |  | ≥1 | 1.01 [0.79-1.30] |  |
| Major bleeding | Apixaban | <1 | 0.69 [0.59-0.80] | 0.51 |
|  |  | ≥1 | 0.77 [0.57-1.04] |  |
|  | Rivaroxaban | <1 | 0.95 [0.81-1.11] | 0.03 |
|  |  | ≥1 | 1.37 [1.01-1.85] |  |
| Intracranial bleeding | Apixaban | <1 | 0.44 [0.31-0.63] | 0.66 |
|  |  | ≥1 | 0.37 [0.18-0.76] |  |
|  | Rivaroxaban | <1 | 0.70 [0.47-1.03] | 0.55 |
|  |  | ≥1 | 0.55 [0.28-1.08] |  |
| CRNM bleed | Apixaban | <1 | 0.70 [0.60-0.82] | 0.59 |
|  |  | ≥1 | 0.77 [0.57-1.05] |  |
|  | Rivaroxaban | <1 | 1.01 [0.93-1.10] | 0.32 |
|  |  | ≥1 | 1.11 [0.94-1.31] |  |
| Net clinical benefit | Apixaban | <1 | 0.85 [0.78-0.93] | 0.75 |
|  |  | ≥1 | 0.88 [0.74-1.05] |  |
|  | Rivaroxaban | <1 | 0.91 [0.81-1.02] | 0.75 |
|  |  | ≥1 | 0.87 [0.68-1.12] |  |
